# Accelerated forgetting of a trauma-like event in healthy men and women after a single dose of hydrocortisone

**DOI:** 10.1038/s41398-022-02126-2

**Published:** 2022-08-31

**Authors:** Vanessa E. Hennessy, Luzia Troebinger, Georges Iskandar, Ravi K. Das, Sunjeev K. Kamboj

**Affiliations:** 1 Clinical Psychopharmacology Unit, Research Dept of Clinical, Educational and Health Psychology, University College London, Gower St, London, WC1E 6BT UK; 2 Department of Anaesthesia and Perioperative Medicine, University College London Hospital, London, NW1 2BU UK

**Keywords:** Human behaviour, Physiology, Predictive markers, Prognostic markers, Psychiatric disorders

## Abstract

Posttraumatic stress disorder (PTSD) is characterised by dysregulated hypothalamic-pituitary-adrenal axis activity and altered glucocorticoid receptor sensitivity. Early treatment with glucocorticoids may reduce PTSD risk, although the effect of such treatment on the aetiologically critical step of traumatic-memory-formation remains unclear. Here we examine the effects of exogenous cortisol (hydrocortisone) in a preclinical model of PTSD, using a factorial (Drug × Sex), randomised-controlled, double-blind design. Healthy men and women (*n* = 120) were randomised to receive 30 mg oral hydrocortisone or matched placebo immediately after watching a stressful film. Effects on film-related intrusions were assessed acutely in the lab, and ecologically using daily memory diaries for one week. We found that participants receiving hydrocortisone showed a faster reduction in daily intrusion frequency. Voluntary memory was assessed once, at the end of the week, but was unaffected by hydrocortisone. Exploratory analyses indicated sex-dependent associations between intrusions and baseline estradiol and progesterone levels. In men receiving hydrocortisone, higher baseline estradiol levels were associated with *fewer* intrusions, whereas women exhibited the opposite pattern. By contrast, progesterone levels were positively associated with intrusions only in men treated with hydrocortisone. The findings suggest that hydrocortisone promotes an accelerated degradation of sensory-perceptual representations underlying traumatic intrusive memories. In addition, while sex alone was not an important moderator, the combination of sex and sex-hormone levels (especially estradiol) influenced hydrocortisone’s effects on involuntary aversive memories. Future well-powered experimental studies may provide a basis for a precision-psychiatry approach to optimising early post-traumatic glucocorticoid treatments that target intrusive memories, based on individual endocrinological profiles.

## Introduction

Persistent, distressing involuntary memories are a transdiagnostic feature of psychological disorders. Such intrusive memories are prototypical of posttraumatic stress disorder (PTSD), a disorder of maladaptive emotional memory-formation characterised by sensory-perceptual hyperamnesia, together with impaired memory for simultaneously encoded contextualised and narrative details of the traumatic event [1].

Given the significant global disease burden associated with PTSD [2] and bottlenecks in capacity to provide efficacious, affordable treatments under current models of care, the development of effective secondary preventative strategies would represent a major advance for trauma survivors globally [3]. Based on the proposed aetiological role of dysregulated trauma memory-formation in PTSD, a theoretically appealing preventative strategy involves restraining the early hyper-consolidation of these memories. Because synaptic consolidation is a brief (<6 hr), protein synthesis-dependent process, commonly used drugs with down-stream protein synthesis-inhibiting effects, such as beta-blockers or NMDA receptor antagonists, have theoretical potential as secondary preventive agents. However, currently, neither meta-analyses of clinical trials [4, 5] nor experimental studies [6] (but see [7]) support use of beta-blockers, and only indirect experimental [8] or cross-sectional [9] evidence on NMDAR antagonists currently exists.

An alternative pharmaco-preventative strategy involves targeting dysregulation of the hypothalamic-pituitary-adrenal (HPA) axis in PTSD [10]. This dysregulation is partly reflected in the low basal cortisol levels found in PTSD patients, a putative risk factor for, rather than consequence of, PTSD [11, 12]. Because low cortisol levels may reflect a reduced capacity for homeostatic restoration of physiological functioning after a traumatic event, several clinical trials have examined the effects of promoting or enhancing such restoration through exogenous administration of glucocorticoids shortly before or after trauma exposure. These provide preliminary evidence for reduced PTSD incidence in glucocorticoid-treated patients [4, 5]. However, because such trials understandably prioritise clinical, rather than mechanistically informative outcomes, and often employ repeated glucocorticoid dosing (therefore affecting more than one memory phase), the extent to which their efficacy depends specifically on modulating consolidation of trauma memories, remains unclear. Moreover, studies of glucocorticoids (particularly, hydrocortisone) have typically been pilot studies with very small sample sizes, raising concerns about the robustness of these findings, while also limiting opportunities to statistically examine biologically important mediators or moderators.

Considering these issues, experimental medicine studies are critical for identifying and optimising PTSD prevention strategies [13]. Such studies allow pathogenic memory phenomena (e.g., distressing involuntary memories [14]) to be modelled and assessed, while limiting the influence of numerous sources of statistical noise common in clinical studies. Two such translational studies have modelled preventative treatment for PTSD with hydrocortisone administered before [15] or shortly after [7] an analogue trauma (a distressing film). Only the latter study demonstrated a reduction in the number of intrusions relative to placebo. Recognising that women are at greater risk of PTSD, the aforementioned translational studies (see also [16]) only recruited women and purposively aimed to constrain natural variation in female sex-hormone levels by recruiting participants who were users of hormonal contraceptives. Indeed, estradiol and progesterone are proposed to have an important role in the aetiology and maintenance of PTSD in women, possibly via dysregulation of associative /extinction learning [17]. Whether they have a similar role in men is not clear.

Given the conflicting previous findings [7, 15] and the need to determine whether men and women are differentially responsive to hydrocortisone treatment, the current pre-registered study tested the time- and sex-dependent effects of hydrocortisone on intrusive memories following an analogue trauma. We also explored the drug- and sex-dependent influence of progesterone and estradiol on intrusions, although this analysis was not pre-registered.

## Materials and methods

The study was pre-registered on the Open Science Framework (osf.io/76yvk). It received ethical approval from the University College London Research Ethics Committee, and all procedures were conducted in accordance with the Declaration of Helsinki; participants provided informed consent. Please refer to the Supplement for further methodological details.

### Participants

Healthy young adult volunteers were recruited via online adverts. Interested participants underwent telephone screening for eligibility. The target sample size (*n* = 120) was based on a power calculation for a simple between groups (Sex × Drug = 4 groups) comparison of total number of intrusions, with an assumption of a large effect size (*f* = 0.4, based on similar pharmacological and behavioural studies; [4, 5]) and *α* = 0.05 and *β* = 0.95. This suggested that a total of *n* = 112 was adequate. Randomisation to group was achieved using a random number generator (see Supplement for details). Eligibility was determined during a telephone screening interview following an initial basic online screening. Participants needed to be healthy adults (18–35 years old). Exclusion criteria included a history of any psychiatric disorder that required treatment, and experience of (a) life event(s) judged at screening to be sufficiently stressful to have resulted in increased risk of PTSD. Women were required to be using a hormonal contraceptive to limit the influence of the menstrual cycle/hormonal fluctuations on intrusive memories [18]. An extensive list of eligibility criteria that were assessed during screening are described in the pre-registration document (osf.io/76yvk). Four participants were excluded (see Supplement) after enrolment, three of whom were replaced, resulting in a final sample of *n* = 119, upon which the main analyses were based. All reported results are from a single (non-replicated) experiment.

At the end of day 1 (the lab session), three participants (males) each reported one adverse response to the capsules. Two complained of headache and one reported feeling “agitated”. However, all were in the placebo group, and none withdrew/were withdrawn due to these mild reactions.

### Drugs

**Fig. 1:**
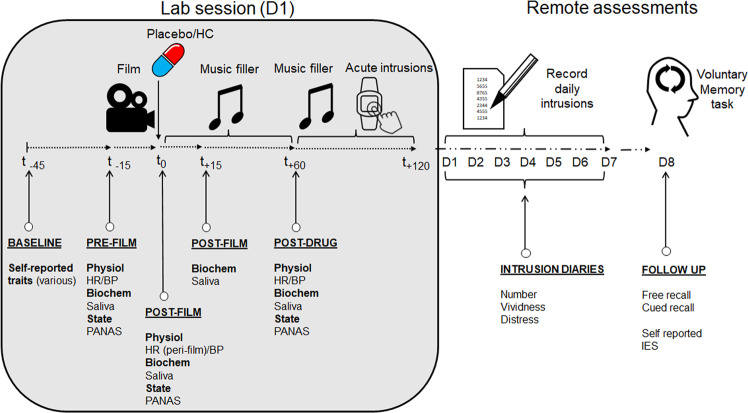
Timeline of relevant experimental procedures. The timeline of the lab session on day 1 (D1) is outlined in the shaded grey box, which indicates the relative timing of in-session procedures (film viewing, drug administration, filler task, button presses in response to acute intrusions) and repeated measures, within-session physiological (HR heart rate, BP blood pressure), subjective “state” (PANAS: Positive and Negative Affect Schedule) and hormonal variables (“Biochem”/Saliva). After drug administration, there was a 60 min drug absorption period and initial music filler task (*t*_0_ to *t*_+60_). The final 60 min of the lab session (*t*_+60_ to *t*_+120_) was a second music filler, although participants additionally recorded spontaneous film-related intrusions in this ‘acute’ drug phase using a wrist-worn monitoring device. Depicted to the right of the lab session box, are the remotely recorded memory procedures, starting at the end of D1 and continuing for the next six days (D2–D7). On D8, participants did not complete a memory diary, but instead completed free and cued recall tasks and the Impact of Events Scale (IES). D2-D8 procedures were conducted remotely.

Hydrocortisone (30 mg) or matched placebo capsules were taken with water under researcher supervision immediately after the trauma film. A 1 hr drug absorption period was then allowed, before post-drug measures were taken (at *t*_+60_; see Fig. 1). Double blinding integrity was tested at the end of day 1, when researchers and participants made concealed, independent treatment guesses. Double blinding was maintained until all data from all participants had been collected.

### Self-report measures

Self-report measures of stable mental health and general psychological characteristics were assessed before the film procedure and drug administration (below and Fig. 1, labelled “Self-reported traits”). Depressed mood was assessed using the Beck Depression Inventory (BDI [19]), anxiety with the Spielberger Trait Anxiety Inventory (STAI [20]), dissociation using the Dissociative Experiences Scale-II (DES [21]), impulsivity with the Barrett Impulsiveness Scale (BIS [22]), habitual responses to emotional experiences using the Emotion Regulation Questionniare-9 (ERQ [23]) and sleep characteristics (average hours of sleep/night) were assessed with the Pittsburgh Sleep Quality Index [24]. Women provided additional information on brand and duration of use of contraception and pattern of menses. General trauma-like symptoms (previous 7 days) were assessed using the 22-item Impact of Events Scale (IES; [25] adapted for the trauma-film [26]).

Changes in state subjective affect from pre- to post-film and post-drug were assessed using the Positive and Negative Affect Schedule (PANAS [27]). The Bodily Symptoms Scale (BSS [28]) was used to assess a range of subjective cognitive and physical sensations (anxiety, depression, memory impairment, palpitations, nausea, emotional numbness, euphoria, drowsiness, muscle tension, headache, concentration, tremor, vertigo, confusion).

### Biological measures

At the same timepoints as the state measures, cardiovascular and endocrine (saliva) samples were taken. Heart rate (HR) was assessed using an ambulatory ECG device (BodyGuard-2, FirstBeat Technologies, Jyväskylä, Finland), and blood pressure (BP) with a standard commercial device (BM40 XL, Beurer UK).

### Trauma film

The trauma video consisted of two scenes depicted extreme violence from the commercial film ‘Irreversible’ (Studio Canal). These were spliced together to form a single narrative with an audio description linking the scenes (see [8]). The film was viewed in a darkened lab on a 17-inch monitor, with audio presented through headphones. These stimuli reliably induce distressing intrusive memories [29].

### Procedure

Key aspects of the laboratory procedure are outlined in Fig.1, with further details in the supplement and published on the Open Science Framework website (osf.io/76yvk; see also [7]). The day 1, in-lab session (starting 1–5 pm) was followed by seven consecutive daily online diaries (starting at the end of day 1), in which participants reported intrusion frequency, content, vividness and distress. All self-report measures were recorded using the online survey tool, Qualtrics, both within and outside of the lab session.

On day 1, after attaching the ECG device, participants completed an inhibitory control task (not reported here) followed by the self-report measures of ‘trait’ mental health and general psychological characteristics (above). Baseline BP, HR, endocrine (saliva), and state subjective affect (PANAS [27]), cognitive and physical characteristics (BSS [28]) were then sampled pre-film (*t*_−15_; see Fig. 1), after which they viewed the trauma film. Drug capsules were then administered, and the state and physiological pre-film measures repeated at the post-film (*t*_0_) timepoint.

A 2-hr music filler period followed, to allow drug absorption (first 60-min: *t*_0_ to *t*_+60_) and recording of acute intrusion (second 60-min: *t*_+60_ to *t*_+120_). The first 60-min of the music filler was interrupted at *t*_+15_ to obtain another saliva sample. For the second 60-min music filler period, participants pressed a response button on a wrist-worn E4 device (Empatica, Boston, MA, USA), whenever they experienced a film-related intrusive memory.

Before leaving the lab, participants were given detailed instructions on how to record intrusive memories. Starting on day 1, participants received an email reminder at 9 p.m. to complete the memory diary as close to bedtime as possible. The interval between the end of the film and the recording of memory events on day 1 (which is assumed to reflect the initial, pre-sleep rehearsal-consolidation period) was similar in the placebo (8.8 ± 2.7 hr) and hydrocortisone groups (9.3 ± 3.4 hr; *t*(117) = 0.86, *p* = 0.393). Reminders to complete the diary were also sent at 9 p.m. on days 2–7. Participants were instructed to record a brief description of each thematically distinct intrusive memory (allowing subsequent verification of film-relatedness of intrusions) and the number of times they occurred. Additionally, participants recorded distress and vividness (1 = ‘not at all’; 5 = ‘extremely’) of each distinct intrusive memory.

Voluntary memory assessment and trauma symptoms (Impact of Events Scale; IES [25], modified for the trauma film [26]) were assessed remotely only on one occasion (day 8). Once completed, participants were debriefed and compensated (£37).

### Statistical analyses

Statistical analyses were conducted using R (R Development Team, 2015) and Stata (Version 17). We tested the following primary pre-registered hypotheses: (i) relative to placebo, hydrocortisone would produce a different rate of reduction in film-related intrusive memories, but voluntary recall measured on day 8, would be unaffected; (ii) sex would moderate drug effects and (iii) post-hydrocortisone cortisol levels would be negatively correlated with intrusion frequency on day 1. Additional exploratory analyses are described below.

Descriptive statistics reported in the text are means with standard deviations. Figures are depicted with either standard errors or 95% confidence intervals. Two-sided tests were used throughout. Mixed ANOVAs with drug and time (three levels: *t*_−15_: pre-film, *t*_0_: post-film, *t*_+60_: post-drug) as independent variables, were used to analyse PANAS ratings, BP, HR and hormone levels (which had a fourth level of the time factor: *t*_−__15_, *t*_0_, *t*_+15_, *t*_+60_; see Fig. 1). Post hoc *p* values are reported with correction for multiple comparisons. Where sphericity was violated, Greenhouse-Geisser correction was used. Because acute (post-film/post-drug) within-lab intrusions (*t*_+60_ to *t*_+120_) were over-dispersed, these were analysed using a negative binomial model. Day 8 variables (free and cued recall and IES) were analysed using univariate ANOVAs.

Generalised linear mixed (Zero inflated Poisson, ZIP) and linear mixed models were used to estimate fixed effects of drug, sex, day and their interactions for diary-recorded intrusion counts, and vividness/distress respectively. Mixed effects models were used to analyse the main hypotheses to account for serial dependencies in the data arising from repeated within-participant assessment. A ZIP model was required for diary count data because of a preponderance of zeros (65%). Validation of the ZIP model via simulation is discussed in the Supplement. Model selection was based on the Akaike Information Criteria (AIC), a function of model fit and complexity. Where AICs of two models were similar, the more parsimonious model was selected.

ZIP analyses testing the relationship between drug condition, sex, and female sex-hormone levels on total dairy intrusion counts were performed separately for estradiol and progesterone (there was no four-way Drug × Sex × Estradiol × Progesterone interaction; *p* = 0.372). The nature of interactions between these variables were not pre-specified and as such, the reported findings are considered exploratory.

## Results

### Participant characteristics

Participant characteristics (Table 1) were in the expected range for normative samples, with average values close to previously published averages from similar studies [7, 15]. Participants and experimenters guessed treatment assignment at chance levels (both 55%; *χ*^*2*^*(1)* ≤ 1.31, *ps* ≥ 0.253) indicating successful blinding.

**Table 1:** Baseline participant characteristics.

	Placebo (*n* = 59^⁋^)	Hydrocortisone (*n* = 60^⁋^)
Sex (♂:♀)	30:29	30:30
Age (yrs)	24.8 (4.5)	24.8 (4.5)
Education (yrs)	16.7 (2.4)	16.5 (2.5)
BDI	4.6 (4.8)	5.1 (4.8)
STAI	32.9 (7.8)	35.4 (7.9)
BIS	36.0 (6.5)	36.7 (6.5)
DES	9.5 (9.2)	9.0 (7.0)
ERQ - reapp	26.5 (4.7)	25.7 (5.5)
ERQ-suppress	/13.9 (5. 6)	14.5 (5.4)
Sleep (hr)	7.6 (0.9)	7.4 (0.9)
BMI (kg/m^2^)	22.8 (3.3)	23.9 (3.2)
Systolic BP (mmHg)	108.6 (13.1)	108.2 (14.0)
Diastolic BP (mmHg)	68.9 (10.7)	69.2 (10.8)
HR (bpm)	72.3 (10.3)	72.5 (9.1)
Cortisol (nmol/L)	3.95 (2.77)	3.63 (2.23)
Cortisone (ng/ml)	4.68 (2.23)	5.59 (2.16)
Progesterone (pg/ml)	9.65 (15.73)	11.68 (13.36)
Estradiol (pg/ml)	3.39 (1.74)	3.47 (1.58)

^⁋^An equal number of men (*n* = 60) and women (*n* = 60) completed the lab session but one (female, placebo group) did not complete the memory diaries and was hence, excluded. The final sample was therefore *n* = 119.

*BDI* Beck Depression Inventory, *STAI* Spielberger Trait Anxiety Inventory, *BIS* Barrett Impulsiveness Scale Sleep, *DES* Dissociative Experiences Scale, *ERQ* Emotion Regulation Questionnaire (*reapp* reappraisal, *suppress* suppression). ‘Sleep’ refers to average hours of sleep per night. In general, men and women showed similar baseline characteristics although there were significant differences in number of years of education (men: *M* = 15.9, *SD* = 2.4, women: *M* = 17.2, *SD* = 2.3; *p* = 0.014) and BMI (men: *M* = 24.4 kg/m^2^, *SD* = 3.2, women: *M* = 22.6, *SD* = 2.9; *p* = 0.014). As previously reported, differences were also found on systolic BP (men: *M* = 113.1, *SD* = 13.0; women: *M* = 103.6, *SD* = 12.3 mmHg, *p* = 0.001 [64]) and on the tendency to suppress emotions (ERQ-suppress: men *M* = 16.0, *SD* = 5.2; women: *M* = 12.3, *SD* = 5.1; *p* = 0.001 [23]). The inclusion of these latter variables as *post hoc* covariates in the analysis of intrusions did not affect the results substantially. Other baseline variables—including estradiol and progesterone levels (see Supplement)—did not differ between men and women (FDR corrected *ps* > 0.05).

### Cardiovascular and subjective changes during the lab session (day 1)

Systolic and diastolic BP, HR and PANAS (negative) all increased from pre- (*t*_−15_) to post-film (*t*_0_; *ps* = 0.001) and recovered to approximately baseline levels by the post-drug timepoint (*t*_+60_; *ps* ≤ 0.041; see Supplement, Figs. S1 and S2). However, there was no main effects or interactions involving drug for any of these variables (*F* values < 1). Although a number of time effects were found for BSS items, no noteworthy drug effects were found (further details in supplement).

### Salivary hormones: cortisol and cortisone

A Time (four levels: *t*_−15_, *t*_0_, *t*_+15_, and *t*_+60_) × Drug mixed ANOVA indicated a robust time-dependent effect of hydrocortisone on salivary cortisol levels (Time × Drug interaction: *F*(1.3, 123.1) = 47.32, *p* < 0.001; *η*_*p*_^*2*^ = 0.33). This reflected supra-physiological levels of cortisol in the hydrocortisone group at 60 min post-drug (*t*_+60_; 86.46 ± 73.56 nmol/L, *c.f*. baseline levels at *t*_−15_ in Table 1; Supplement, Table S1), which were significantly higher than each of the previous three timepoints (i.e., *t*_+15_, *t*_0_, and *t*_−15_; all *ps* < 0.001). By contrast, salivary cortisol levels in the placebo group at *t*_+60_, (2.97 ± 3.59 nmol/L) did not differ from any of the previous timepoints (*ps* > 0.99). The effect size of ∆Cortisol from *t*_−15_ to *t*_+60_ in the hydrocortisone group was *d*_*(within)*_ = 1.18. For comparison, uncontrollable and/or social-evaluative psychological stressors yield an effect size of ~0.4 [30]. The absolute change in cortisol reported here following hydrocortisone is also many times larger than that found in studies using the standard experimental physical stressor employed in experimental studies involving stress-induction (the cold pressor; [31]). These supra-physiological cortisol concentrations following hydrocortisone are however consistent with previously reported effects of hydrocortisone assessed using immunoassay techniques [7, 32].

Similar to cortisol, salivary cortisone, a purportedly more accurate measure of free serum cortisol levels, uncontaminated by oral hydrocortisone [33], showed a significant Time × Drug interaction (*F*(1.1,101.6) = 102.04, *p* < 0.001; *η*_*p*_^*2*^ = 0.52). Again, cortisone levels at *t*_+60_ in the hydrocortisone group (28.35 ± 16.23 ng/ml, *c.f*. 5.43 ± 2.08 at *t*_−15_; Table S1) were significantly higher than the three previous timepoints (*ps* < 0.001) whereas cortisone at t_+60_ in the placebo group (4.36 ± 2.74 ng/ml) did not differ from cortisone at *t*_+15_, *t*_0_, or *t*_−15_ (*ps* > 0.99).

### Intrusive memories

#### Acute post-film intrusions

In the 60 min period starting 60 min after drug administration (i.e., *t*_+60_ to *t*_+120_; see Fig. 1), instantaneously recorded intrusions did not differ significantly in the two drug groups: *IRR* = 1.19, *SE* = 0.30, z = 0.69, *p* = 0.491 (for reference, the placebo group experienced 7.38 intrusions (SE = 1.31) in that 60 min period). This suggests that involuntary retrieval of trauma-like memories in the post-encoding period was unaffected by the rapid, non-genomic effects of hydrocortisone.

Per the pre-registered analysis plan (and previous findings [7]) we examined the association between post-drug cortisol levels in the hydrocortisone group and (sub-acute) day 1 intrusions (from diary entries at the end of day 1). We found no significant linear or quadratic relationship in the hydrocortisone group as a whole (*p*s > 0.3). Given the previously observed significant correlation in women [7], we also conducted separate correlations in men and women in the current sample. However, this also did not reveal a significant association in either sex, although descriptively the association was more negative in women (*r* = −0.181, *p* > 0.3) than men (*r* = −0.043, *p* > 0.8).

### Intrusive memories across days

#### Intrusion counts

In line with our pre-registered hypotheses, we tested the effects of time, sex and drug on intrusion counts. Backwards selection based on AIC values showed that neither the three-way interaction, nor the two-way interactions involving sex (Sex × Drug and Sex × Time) were important. In the optimal model, as judged by AIC, sex had minimal effect on model fit. Since the models with and without the sex main effect had similar AIC values we omitted the sex effect. Therefore, the optimal ZIP model contained a drug, time, and Drug × Time interaction, plus a random intercept for participant.

**Fig. 2:**
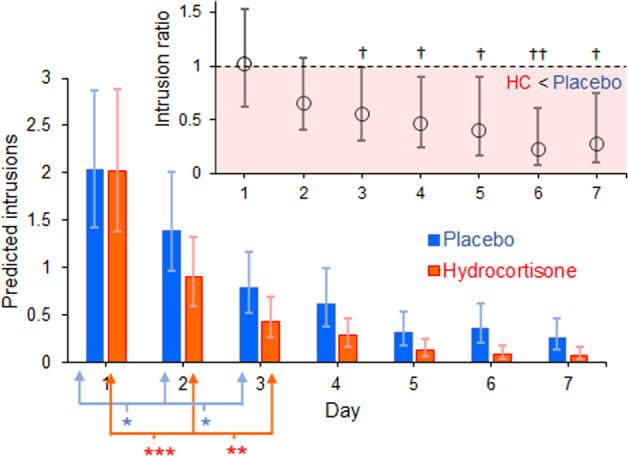
Accelerated reduction in intrusive memories following hydrocortisone. Main panel: predicted mean (95% CIs) number of intrusions on days 1–7 in the placebo (blue bars) and hydrocortisone (red bars) groups. Significant sequential reductions between days for each drug (*p < 0.05; **p < 0.01, ***p < 0.0001) are indicated. Inset: ratio of hydrocortisone intrusion counts relative to placebo, with values in the shaded region indicating lower hydrocortisone counts. Simulated ratios were skewed, accounting for the asymmetric 95% CIs. Upper bounds of 95% CIs do not cross 1 (dotted line) from day 3 onwards, indicating fewer intrusions in the hydrocortisone (HC) group (^†^*p* ≤ 0.05; ^††^*p* < 0.01).

This model indicated a faster decline in intrusions in the hydrocortisone group (Fig. 2; Day × Drug interaction: (*χ*^*2*^*(6)* = 27.40, *p* < 0.001). Specifically, there was a larger mean difference between days 1 and 2 in the hydrocortisone group (*b* = 0.81, *SE* = 0.13, *t(817)* = 6.20, *p* < 0.001) relative to placebo (*b* = 0.38, *SE* = 0.13, *t(817)*=3.01, *p* = 0.043). Similarly, larger reductions between days 2 and 3 were found with hydrocortisone (*b* = 0.73, *SE* = 0.208, *t(817)*=3.52, *p* = 0.008) relative to placebo (*b* = 0.57, *SE* = 0.17, *t(817)* = 3.40, *p* = 0.0123). No other sequential day effects were found in either drug group (all *ps* > 0.3). Note, that the difference (*beta*) values were calculated from log coefficient estimates (see Supplement; Tables S2 and S3) rather than the original count units, and hence do not correspond to differences between bars displayed in Fig. 2.

Between-group comparisons at each level of day confirmed a differential effect of drug across days. As shown in the inset of Fig. 2, the ratio of intrusions in the hydrocortisone to intrusions in the placebo group showed a non-significant difference relative to a 1:1 ratio (the dashed line) on day 2 (*b* = −0.43, *SE* = 0.25, *t(817)* = 1.72, *p* = 0.087). The drug group difference was marginal (*p* = 0.05) on day 3, and significant from day 4 onwards (*ps* ≤ 0.0362).

### Distress and vividness

**Fig. 3:**
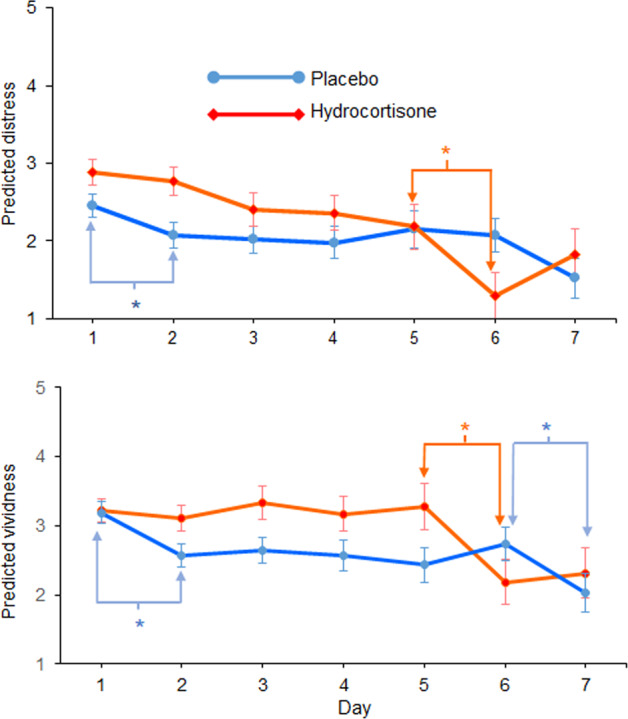
Intrusion-related distress and vividness levels. Predicted average (±SE) day by drug intrusion-related distress (top panel) and vividness (bottom) ratings for placebo (blue lines) and hydrocortisone (red lines) *p < 0.05.

Linear mixed-effects models were used to test fixed effects of sex, day and time, and their interactions on distress and vividness. The best fitting model containing random per-participant intercepts, main effects of day and drug and the Day × Drug interaction, was not substantially improved when sex was included as a factor. Moreover, sex was not a significant moderator, so as above, it was excluded from the final model. This model indicated a significant interaction between drug and day (*χ*^*2*^*(6)* = 16.58, *p* < 0.011), reflecting a more complex pattern of effects relative to the count data. Specifically, reduction in distress was non-monotonic and there were no sequential day effects in the hydrocortisone group until day 5, when distress dropped precipitously and significantly (∆Distress _(Day 5→6)_ = 0.9, *SE* = 0.4, *z* = 2.40, *p* = 0.017; Fig. 3). By contrast, in the placebo group, there was a smaller, but nonetheless, significant early reduction in distress ratings (∆Distress _(Day 1→2)_ = 0.4, *SE* = 0.2, *z* = 2.21, *p* = 0.027), but no subsequent sequential difference between days. Alternatively, when distress ratings were weighted by the number of intrusions (distress load), there was no indication of a Day × Drug interaction (*χ*^*2*^*(6)=*1.72, *p* = 0.94). However, such weighting may have been strongly influenced by the large number of zero counts later in the week, diluting any group differences.

The model of best fit for vividness included a random per-participant intercept and day, sex, drug and Day × Drug terms. As shown for distress there was also a Day × Drug interaction for vividness (*χ*^*2*^*(6)* = 14.35, *p* = 0.0259), again reflecting somewhat sporadic change across days in the two groups. A main effect of sex was also found, with women showing higher overall vividness (*χ*^*2*^*(1)* = 5.24, *p* = 0.022). Like the distress ratings, an early (day 1→2) significant reduction in vividness was seen in the placebo (∆Vividness _(Day 1→2)_ = 0.6, *SE* = 0.2, *z* = 3.44, *p* = 0.001; Fig. 3) but not hydrocortisone group. There was also a significant reduction later in the week (day 6–7) in the placebo group (∆Vividness _(Day 6→7)_ = 0.7, *SE* = 0.3, *z* = 2.11, *p* = 0.035), but a larger reduction in the hydrocortisone group from day 5 to 6 (∆Vividness _(Day 5→6)_ = 1.1, *SE* = 0.4, *z* = 2.54, *p* = 0.011), paralleling the effect seen on distress ratings.

### Day 8 effects: Voluntary trauma-film recall and PTSD-like symptoms

Free and cued recall performance on day 8 did not differ between the two drug conditions. In the free recall task, Participants in the placebo group recalled *M* = 36.6 (*SD* = 18.3) versus *M* = 37.4 (*SD* = 14.9) ‘idea units’ among participants in the hydrocortisone group (*F*(1,117) = 0.075, *p* = 0.785). Cued recall performance was also very similar in the placebo (*M* = 9.0, *SD* = 2.4)) and hydrocortisone groups (*M* = 8.9, *SD* = 2.2; *F*(1,116) = 0.057, *p* = 0.812). There was no differential increase in negative affect (*F*(1,116) = 2.85, *p* = 0.094), or decrease in positive affect (*F*(1,116) = 0.02, *p* = 0.898) from pre- to post recall in the two drug groups. There was also no effect of hydrocortisone on total IES scores (placebo: *M* = 14.3, *SD* = 11.9; hydrocortisone: *M* = 15.12, *SD* = 12.8; *F*(1,117) = 0.128, *p* = 0.721). None of the subscale differed between the two groups (*F* values ≤ 0.41, *p*s ≥ 0.521).

### Association between intrusive memories and female sex hormones

**Fig. 4:**
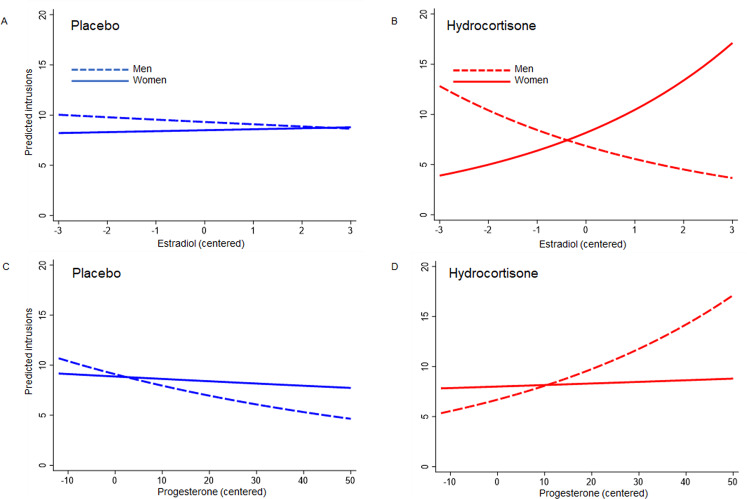
Hormone-by-sex-by-drug interactions. The association between intrusions and baseline mean-centred estradiol levels (**A**, **B**) and between intrusions and baseline mean-centred progesterone levels (**C**, **D**). Dashed lines = men; solid lines = women. Blue = placebo; Red = Hydrocortisone.

As can be seen in Fig. 4, at mean levels of estradiol and progesterone, men and women did not differ substantially on total intrusion counts (NB, hormone levels are mean centred in Fig. 4). However, sensitivity to hydrocortisone’s effects appeared to depend on the interaction between sex and sex-hormone levels. Considering the effects of estradiol first, we found that intrusions were jointly determined by estradiol levels, sex and drug group (three-way interaction: *b* = 0.420, SE = 0.085, *z* = 4.96, *p* < 0.001). Unlike the placebo condition (Fig. 4A; Sex × Estradiol: *b* = 0.036, SE = 0.054, *z* = 0.67, *p* = 0.504), hydrocortisone-treated men showed fewer intrusions as estradiol levels increased (dashed red line), whereas women showed the opposite pattern (Fig. 4B, Sex × Estradiol, *b* = 0.455, SE = 0.065 *z* = 7.00, *p* < 0.001; solid red line). The moderated relationship between progesterone and intrusion counts was less pronounced, but still significant (Progesterone × Sex × Drug interaction: *b* = 0.028, SE = 0.011, *z* = 2.61, *p* = 0.009). Again, the significant Sex × Progesterone interaction resided in the hydrocortisone group (*b* = −0.017 SE = 0.007 *z* = 2.28, *p* = 0.022; Fig. 4D) rather than the placebo group (*b* = 0.011, SE = 0.008, *z* = 1.41, *p* = 0.158; Fig. 4C). In hydrocortisone-treated women, progesterone levels did not appear to influence intrusion counts (solid red line in Fig. 4D), but in contrast to the relationship seen with estradiol, higher levels of progesterone were associated with higher levels of intrusions in men (Fig. 4D; dashed red line).

## Discussion

In this study, we tested the effects of hydrocortisone on a transdiagnostic symptom of psychopathology—intrusive memories—and examined the conditions that might affect its efficacy in an experimental medicine model of the onset and prevention of PTSD. In line with our first pre-registered hypothesis, we found that compared to placebo, hydrocortisone produced a different (accelerated) decline in intrusive memories. This was reflected in larger day 1→2 and day 2→3 reductions in intrusion counts in the hydrocortisone group, as well as differential placebo *vs*. hydrocortisone effects on each day, starting on day 2 and becoming (marginally) significant from day 3. These effects on *occurrence* of intrusive memories were dissociable from effects on *distress* and *vividness*, which showed a non-monotonic decline, with drug group differences observed at the beginning and end of the week. Divergent effects on the rate of reduction between intrusions, vividness and distress have been reported previously studies [7] and potentially reflect dissociable effects of hydrocortisone on memory, affective and visuospatial cognitive systems. Overall, the findings are somewhat consistent with our previous results with hydrocortisone (and propranolol [7]), although the faster decline in intrusions following hydrocortisone has closer parallels to our previous study with nitrous oxide [8].

The rationale for the current study was partly based on incidental observations in patients receiving ICU care for hyperdynamic septic shock, who showed lower incidence of PTSD when treated with hydrocortisone [34, 35]. Subsequent small-scale placebo-controlled trials were consistent with these early observations (reviewed in ref. [4]). Our findings suggest that hydrocortisone’s effects on general PTSD incidence/symptomatology in trauma-exposed individuals might reflect a more specific effect on the sensory-perceptual *memory processes* underlying re-experiencing symptoms.

These findings can be interpreted through the lens of an influential clinical theory of PTSD: the dual representation theory, which proposes that sensory-perceptual involuntary memories on one hand, and context-rich voluntary memories on the other, represent the expression of dissociable memory systems [36] The theory implies that intact functioning of the contextualising memory system facilitates recovery after trauma-exposure by enabling the formation of a more complete and/or coherent representation of the traumatic event through integration of relatively fragmented information stored in the sensory-perceptual memory system [37]. As such, ideal memory-therapeutic strategies used in secondary prevention of PTSD should selectively affect sensory-perceptual memory functioning whilst sparing voluntary-contextualising memory [38].

Existing glucocorticoid-memory research has largely focused on cued-associative memory (fear conditioning/extinction) or voluntary episodic-verbal (word lists) memory and has generally demonstrated *enhanced* long-term memory performance with elevated cortisol during encoding/consolidation (see [39] for a fear-extinction example, and [40] for an example with verbal memory). As such, apart from indirect evidence from clinical trials mentioned previously (which assessed gross symptomatology—including re-experiencing—rather than memory functioning specifically), there does not seem to be a strong a priori basis upon which to expect a *selective ‘impairment’* of sensory-perceptual memory consolidation by glucocorticoids. The current demonstration of an accelerated decline in intrusions following hydrocortisone treatment during the putative period of synaptic consolidation is not a sufficient basis to claim an effect on consolidation, let alone a *specific* effect on sensory-perceptual memory. Below, we discuss how our findings might be reconciled with the complex and varied effects of glucocorticoids on human memory.

Firstly, the larger reduction in intrusions from day 2 (i.e., the day after the trauma video) may partly reflect a quadratic relationship between cognitive performance and cortisol levels. In particular, supra-physiological levels of cortisol at t_+60_ might represent the rightmost part (i.e., the ‘impairment arm’) of an inverted U-shaped dose-response curve. Indeed, studies that examined varying levels of endogenous cortisol/doses of hydrocortisone have demonstrated impairment at high levels/doses [41–44]. Whether the current findings reflect a specific reduction in (sensory-perceptual) memory *consolidation* rather than a generalised cognitive impairment at high concentrations of cortisol is unclear. However, it should be noted that the lack of effect of hydrocortisone on voluntary, contextualised-verbal memory performance in the current study is not consistent with a global impairment in cognitive functioning.

Secondly, in a previous study [7] we suggested that hydrocortisone’s delayed effects on intrusions might have reflected earlier acute effects on spontaneous retrievals, that ordinarily support consolidation [45]. Indeed, hydrocortisone’s well-established impairing effects on retrieval of long-term memories [46] have also been observed shortly after encoding (i.e., before the memory was fully consolidated; [47, 48]) and this could therefore represent an indirect route to impaired early (synaptic) consolidation. We tested this possibility in the current study by assessing intrusions acutely (i.e., 1 hr after the trauma video) but found no group differences. Alternatively, Miller and colleagues (2015) showed that hydrocortisone hastened the decay of iconic memory traces [49]. If iconic or similar short-term memory systems act as a buffer for fleeting, internally generated visual images, degradation in their functioning by hydrocortisone might also degrade the maintenance of sensory-perceptual representations underlying distressing intrusions. This might, again, indirectly affect early consolidation and explain hydrocortisone’s later (from day 2) effects on involuntary (sensory) memories.

On the other hand, the delayed emergence of hydrocortisone’s effects could have relied upon systems-level reorganisation of memory rather synaptic consolidation processes. Given the >24 hr delay before group differences were evident, sleep might also have played a role in determining hydrocortisone’s effects on intrusions [50]. Glucocorticoid-treated rodents in an animal model of traumatic stress also show delayed effects on plasticity in structures involved in emotional learning [51, 52]. It is possible that hydrocortisone’s effects on intrusions also reflects delayed protective effects on brain regions involved in memory contextualisation, while constraining plasticity in structures involved in maladaptive emotional learning (e.g. [53],).

Finally, elevated cortisol can have generalised acute effects on emotional and cognitive processing, which unfold over the course of several hours. These are reflected in, for example, improved prefrontal functioning during neutral cognitive tasks [54]. These generalised effects might also include enhanced prefrontal activation during emotion regulation following exposure to negatively valenced stimuli [55]. As such, hydrocortisone’s effects on intrusions might be an indirect consequence of enhanced and temporally extended emotion regulation.

Despite the above discussion about the role of cortisol in memory effects, it should be noted that unlike our previous work in a women-only sample [7], we did not observe the hypothesised negative association between post-hydrocortisone cortisol levels and intrusion frequency on day 1. It is unclear if the relatively minor methodological differences between our current and previous [7] study are sufficient to explain the divergent effects. One potential methodological factor was the use of a mixed sample of men and women in the current study. However, the cortisol-intrusion association was also non-significant when women were analysed separately, although the direction of the effect was as predicted. It is possible that this association is only present in women, although the true effect might be too small to have been observed in the current, relatively small sample.

In addition to the time (i.e., day-) dependent effects of hydrocortisone described above, we also examined its dependence on sex. Sex is an established determinant of the effects of stress on memory [56], the endocrine response to trauma [57], and the effects of hydrocortisone on emotional memory [58]. Sex might also moderate protection against PTSD following dexamethasone treatment [59]. However, our data did not support the pre-registered Sex × Drug interaction hypothesis. On the other hand, our exploratory analyses did show associations between estradiol and progesterone levels and total intrusions that were dependent upon drug condition and sex.

Existing research on the effect of estradiol and progesterone on intrusive memories is limited and the findings are inconsistent [17]. We are aware of only one study that examined the effect of sex in relation to the association between estradiol and progesterone and intrusive memories (although see [60], which tested the moderating role of these hormones on the likelihood of developing chronic PTSD in both sexes). That study [61] reported a positive association between estradiol and intrusions in women, and a negative relationship in men. This is the same directional pattern as our findings with hydrocortisone. However further comparisons with that study are complicated by the fact that the authors [61] analysed their low-frequency count data as if it was continuous and did not present details on any interactions between their ‘arousal group’ factor (i.e. high and low endogenous cortisol groups) and sex-hormone levels. Regardless of the limited previous relevant research on sex-by-sex-hormone interactions in PTSD and memory functioning, our findings provide a basis for future *confirmatory* studies that could improve our understanding of individual differences in memory effects of glucocorticoids. Despite only recruiting contraceptive-using women, our study found considerable variations in background estradiol levels in these participants (see Supplement). This might suggest that naturally cycling women, who show large *intra-individual* variations in oestrogen levels, would exhibit differential sensitivity to hydrocortisone dependent on menstrual cycle phase. In addition, our results suggest that, in men, it may be possible to pharmacologically augment the effects of hydrocortisone on intrusive memories by co-administering estradiol.

### Limitations

We acknowledge a number of limitations of the current study. The trauma-film paradigm is, by definition, an analogue procedure designed to elicit intrusions in an ethically acceptable way in healthy people. One concern is that the resulting intrusions do not mirror the phenomenon seen following real-life traumas, especially in terms of the number of intrusions. Intrusion counts were indeed quite low in the current study, although they were by no means atypical relative to similar experimental studies. In fact, they were also not very different from intrusion frequencies seen in clinical participants [62]. Nonetheless, the extent to which findings from an analogue ‘secondary prevention’ study in healthy participants translate to clinical participants remains unclear (although our findings are consistent with meta-analyses of small-scale clinical trials of hydrocortisone treatment in PTSD [4, 5]).

### Concluding comments

Our findings are consistent with a number of small-scale clinical trials of hydrocortisone on PTSD prevention. They also suggest that hydrocortisone’s preventive efficacy in PSTD may be dependent upon an accelerated degradation of the sensory-perceptual representations that underlie intrusive memories. The findings also raise a number of questions, such as whether the observed day-dependent effects of hydrocortisone are specific to sensory-perceptual involuntary memories. In particular, would voluntary memory show a similar day-dependent effect (in the absence of a main effect of drug) if it was assessed repeatedly and matched to involuntary memory on various dimensions at test [63]? The current study also supports the notion that estradiol and progesterone are important determinants of memory functioning relevant to PTSD and other psychiatric conditions. They additionally highlight the importance of assessing sex-hormone levels as potential prognostic indicators of treatment response. Finally, they point to a potential modulating role for ‘ovarian’ hormones in both women *and* men. Given that few other studies have investigated the role of female sex hormones in memory functioning in both sexes, it will be important to replicate the current findings and potentially extend them by examining the effects of simultaneously manipulating sex-hormone levels.

## Supplementary information

Hennessy et al (2022) SUPPLEMENT
