# Supplementary material for: Accelerated forgetting of a trauma-like event in healthy men and women after a single dose of hydrocortisone
Source: Transl Psychiatry. 2022 Aug 31;12:354. doi: 10.1038/s41398-022-02126-2 (PMC9433412; doi:10.1038/s41398-022-02126-2)
Supplement: Supplementary file 1 — Hennessy et al (2022) SUPPLEMENT [file 41398_2022_2126_MOESM1_ESM.rtf]

Hennessy et al (2022) SUPPLEMENT
Accelerated forgetting of a trauma-like event in healthy men and women after a single dose of hydrocortisone

Supplementary Materials and Methods

Additional procedural details are provided on the Open Science Framework (osf.io/76yvk).

Participants
Eligibility was determined during a telephone screening interview following an initial basic online screening. Participants needed to be healthy young adults (18 -35 years old), agreeing to complete the first three days and at least five of seven daily online memory-diary entries for the week following the trauma video. Those with a self-disclosed history of psychiatric disorder were excluded at screening, as were those who did not pass the diagnostic screening for history of PTSD. An additional requirement for women was ongoing (>1 month) and verifiable use of a hormonal contraceptive. Such treatment tends to stabilize ovarian hormone concentrations at follicular phase levels[1] hence limiting the influence of menstrual cycle-related fluctuations on intrusive memories [2]. In addition, women taking hormonal contraceptives have been found to have similar estradiol and progesterone levels to men [3]. 

An equal number of men (n=60) and women (n=60) completed the lab session. Of the original n=120, three participants were replaced: two men assigned to the hydrocortisone group asked to withdraw during the lab session, prior to drug administration because they found the contents of the film too aversive. Another participant (female, placebo) was found to have taken part in an unrelated pharmacological study during this study (between day 1 and 7). After attending the lab session on day 1, one additional participant (female, placebo) failed to complete the diary entries. She could not be replaced due to timing (the COVID-19 pandemic-lockdown had just started in the UK). As such, the final sample was n=119.

Drugs and randomization
Hydrocortisone (Auden Mckenzie Pharma Division, Ruislip, UK) and placebo (skimmed milk powder) were prepared in identical gelatine capsules (two capsules per participant; see [5]. Treatment envelopes containing hydrocortisone or placebo capsules were labelled with participant id, and both participant and experimenter were blind to drug condition. Allocation to drug group was based on an online random sequence generator (https://www.random.org/sequences/), which produced a non-repeating, randomly ordered sequence of integers (1-120), each corresponding to a unique participant id. This was copied to a spreadsheet and each number in the random sequence was alternately assigned to one of the two drug conditions - placebo or hydrocortisone – and, in a separate column, to male or female. As such, there was an equal number of men and women assigned to placebo and hydrocortisone conditions before the sequence was ordered from 1-120. Upon entry, depending on their sex, participants were assigned to the next unique id. At the end of the lab session on day 1, participants entered a treatment guess (placebo or hydrocortisone) directly into the computer survey programme. Their entry was not visible to the researcher, who subsequently entered an independent treatment guess into the computerized survey.


Memory assessments

Intrusive memories
After viewing the film and reading a description of the nature of intrusive memories, participants recorded momentary intrusions for 60 min on an E4 device (Empatica, Boston, MA, USA), starting after the 1 hr drug absorption period (i.e. t+60 to t+120) during a second filler (music) task period. Thus, while listening to a standard set of classical music pieces and periodically rating these for pleasantness (sham task), participants were instructed to press the response button on the E4 once for every occurrence of an involuntary memory relating to the film (active task). This acute period of memory monitoring was intended to capture post-encoding memory retrieval which may contribute to early consolidation differently in the two drug groups (retrieval is an important mechanism in consolidation [17]). Any early difference in groups would be expected to reflect non-genomic effects of hydrocortisone/cortisol [6]. 

Before leaving the laboratory, participants were given detailed instructions on how to record intrusive memories over the following week (see [5, 7]; see also under 'Data collection procedures' on osf.io/76yvk). The definition of intrusive memories provided to participants emphasized their involuntary nature and distinguished between sensory and verbal mental events. Based on the descriptions provided in the memory diaries, all recorded intrusions conformed to the definition of sensory-perceptual memories. 

Starting at the end of day 1, and on each subsequent day at 9 p.m., participants received an email reminder to complete the memory diary as close to bedtime as possible. On day 1, the interval between the end of the film and completion of the memory diary (which is assumed to reflect the initial, pre-sleep rehearsal-consolidation period) was similar in the placebo (M=8.8 ± SD=2.7 hr) and hydrocortisone groups (M=9.3 ± 3.4 hr; t(117)=0.86, p=0.393). For the diary entries, participants were instructed to record a brief description of each thematically distinct intrusive memory (allowing subsequent verification of film-relatedness of intrusions) and the number of times they occurred. Additionally, participants recorded distress and vividness of each distinct intrusive memory (1 = 'not at all'; 5 = 'extremely'). 

Voluntary (recall) memory
Voluntary memory was assessed on one occasion only (day 8). For the free recall task, participants were asked to recall “as much information and detail as possible including information about where things happen, when they happen, who they happen to, what the people and scenes look like, etc.”, typing this directly into a text box with no time limit. Performance was determined by counting the number of recalled 'idea units.' After the free recall task, participants provided open text responses to 19 questions about events in the two clips (cued recall). These questions related to contextual aspects of the narrative (i.e., who does what to whom, and when and where events take place). There was no time limit for either recall task. Every participant's free and cued recall task performance was independently scored either 0 (inaccurate), 0.5 (partially accurate), or 1 (accurate) by two treatment-blind researchers. High interrater agreement was achieved (>90%) in scoring both tasks with disagreements resolved through discussion. Positive and negative affect (PANAS, [8]) ratings were taken before and after the memory recall tasks.


Salivary hormone levels
Steroid hormone levels were determined as part of a hormone panel from saliva samples taken pre-film (t -15; see Figure 1), immediately post-film (t 0), 15-min post film (t +15) and 1-hour post drug (t +60). In addition to salivary cortisol, cortisone levels were also obtained as a more accurate reflection of free serum cortisol levels that are less likely to be contaminated by oral hydrocortisone administration [9]. Saliva was collected in salivettes as passive drool and stored at -80℃. Cortisol, cortisone and progesterone levels were analysed using liquid chromatography-tandem mass-spectrometry (LC/MS) as described in detail elsewhere [10]. Estradiol levels were measured using a commercially available chemiluminescence immunoassay with high sensitivity (IBL International, Hamburg, Germany). The intra and interassay coefficients for estradiol were below 11%.

Cardiovascular measures
Sympathetic activation in response to the film was assessed via changes in blood pressure (BP; BM40 XL, Beurer, UK) and heart rate (HR; BodyGuard-2, FirstBeat Technologies, Jyväskylä, Finland) sampled pre-, and post-film (as well as post-drug) as outlined elsewhere [5]. Event markers were added periodically. These corresponded, for example, to the start 
(t -15) and end of the film (i.e. t0), and end of the 1 hr drug absorption period (t+60), and were used to identify 5-min epochs for use in obtaining average HR.

Procedure: additional details 

Day 1: To minimise the influence of diurnal fluctuations in circulating cortisol (e.g., [11] which can affect learning and determine the outcome of learning-based treatments [12] testing started between 1 p.m. and 5 p.m.  Upon arrival, participants confirmed fasting (except for water) for the past ≥2 hr, and abstinence from alcohol/recreational drugs for ≥24 hr (and intention to do likewise for the 24 hr after the session). Women provided additional information on brand and duration of use of contraception and pattern of menses. ECG electrodes were attached to the torso, either side of the midline (Bodyguard 2, Firstbeat; see [13] for further details). A simple EEG headset with dry electrodes (DREEM band, Rhythm, Paris, France) was worn during the procedure, although this data was not used. Participants first completed an inhibitory control (anti-saccade) task whilst head movements were minimised using a chin rest. They then provided details about their sleep patterns and completed 'trait' questionnaires as outlined in main text. HR was recorded continuously and sampled 5 min before the film. 

After these measures, participants viewed the trauma film while HR was continuously sampled, immediately after which, drug capsules containing either hydrocortisone or placebo were swallowed by the participant with water under experimenter supervision. BP, saliva samples and subjective state measures were then repeated. These immediately post-film (t0; BP, salivary hormones, subjective state) measures were intended to capture the acute stress response to the film.  Note the t0 HR period corresponded to the last 5 min of the film.

Statistical analyses
As expected, there was very little missing data on the main (intrusion-related) outcomes (two missing entries of 833 possible entries for the included n=119 participants; <0.3% missing). The missing counts were replaced by the value for that participant from the next day's diary entry (referred to as 'next observation carried back' in the missing data section of the preregistration document). Per the pre-registered analysis plan, outliers were defined as having a standardized absolute value (Z score) > 3. These outlying values were winsorised to 1+ largest non-outlier. This applied to 11 individual data points for the intrusion frequency data (1.3%), seven of which came from a placebo participant, and four from hydrocortisone participants. Eight of these data points were from days 3-7. Baseline characteristics were compared between men and women and between different contraceptive using women, using independent samples t-tests, with Benjamini-Hochberg false discovery rate (FDR) adjustment applied to p values. Equality of variance assumptions were found to hold and therefore no adjustments to dfs or p values were made. Generally, men and women had similar baseline characteristics, except for years of education, BMI, systolic BP and suppression, as outlined in the footnote to table 1.
In all analyses, the within-subjects factor was either the within lab-session 'timepoint' (with 3 or 4 levels, depending on the variable) or 'day' (7 levels: days 1-7). Drug group was the key between-subjects independent variable and sex, the primary between-subjects moderator. Differences in cell counts in a 2 x 2 table of Assigned Condition x Guessed Condition were analysed using a Chi squared test.  
Within-session changes in BP, HR, cortisol/cortisone and PANAS ratings were analysed using repeated measures ANOVAs. Violations of sphericity were corrected by adjusting dfs and p values. P values associated with post hoc pairwise comparisons are reported with Bonferroni correction. For intrusion outcomes where day was a factor, general or generalized linear mixed models (GLMM) were used to estimate fixed effects of drug, sex, day and their interactions using the glmmTMB package. To model the serial dependency of data-points from the same person (across days), 'participant' was included as a random factor in the analyses. As is typical in trauma film research [5, 7], intrusion data were over-dispersed (dispersion statistic: 1.18, p<0.0001). There was also a preponderance of zeros (65%), suggesting that the appropriate modelling approach was the zero inflated Poisson (ZIP) GLMM. Simulation with 1000 datasets [14] confirmed overdispersion (p<0.0001) and zero inflation (>95% of simulated samples contained fewer zeros than our data; p=0.032), validating the ZIP model. Model assumptions were checked by plotting residuals against predicted values. No problematic residuals were detected. Confidence intervals for estimates presented for the mixed effects ZIP analysis were obtained using the procedure outlined in [15]. Descriptive statistics for other (continuous) variables below are presented as mean differences (∆) or means + either SE or SD, as indicated. 
Supplementary Results
Cardiovascular and subjective changes during lab session (day 1) 
Time (pre-film: t-15, post-film: t0, post-drug: t+60) by drug (placebo, hydrocortisone) repeated measures ANOVAs indicated that changes in diastolic and systolic BP over time were not moderated by drug; there was also no main effect of drug (interaction and main effect F values <1). Main effects of time were found for both systolic (F(2, 234)=7.05, p=0.001) and diastolic BP (F(2, 234)=5.48, p=0.005; Fig S1A). This reflected an increase in systolic BP from pre- to post-film (∆Systolic t-15t0=+3.2,  p =0.001), followed by recovery within an hour (∆Systolic t0t+60 =-2.8, p=0.005). A similar pattern (increase BP followed by recovery) was found for diastolic BP (∆Diastolic  t-15t0 =+2.7, p=0.009; ∆Diastolic t0t+60 =-2.0, p=0.041; Fig S1B). 


Figure S1. A: Mean + SE systolic blood pressure for placebo (blue) and hydrocortisone (red). Time points 1,2 and 3 are t-15 (pre-film), t0 (post-film) and t+60 (post-drug) respectively. B: Mean + SE diastolic blood pressure.

A		
B		

HR also showed a main effect of time (F(2,224)=38.93, p<0.001; çp2=0.26), but no main/interaction effect of drug (F values<1). The time effect for HR, resembled the pattern for systolic BP: a t-15 to t0 increase (∆HR t-15 t0=+2.2, p<0.001), followed by recovery between (∆HR t0 t+60 =-4.6, p<0.001; Fig S2).

Figure S2: Mean heart rate (+ SE) in beats per minute across timepoints for placebo (blue) and hydrocortisone (red). Time points 1,2 and 3 are t -15 (pre-film), t 0 (peri-film) and t +60 (post-drug) respectively


Paralleling the changes in the above indices of sympathetic arousal, negative affect showed a main effect of time (F(1.3, 155.8)=130.32, p<0.001), but no main/interaction effects involving drug (F values <1). The effect of time reflected an increase in negative affect from pre- to post-film (∆PANAS(negative) t-15t0=+6.8, p<0.001) followed by recovery at t+60 (∆PANAS(negative) t0t+60=-7.1, p<0.001). The time effect (and absence of drug effects F<1) on positive affect (F(1.3, 155.8)=64.95, p<0.001) was in the opposite direction from  pre- to post-film (∆PANAS(positive) t-15t0=-4.9, p <0.001) but unlike negative affect and the cardiovascular indices, there was on recovery within 1 hr (∆PANAS(positive) (t0t+60): -0.1 (p>0.99). 

Supporting the notion that changes in cardiovascular activity and negative affect had a common underlying cause, ∆PANAS(negative) t-15t0 was positively correlated with ∆Systolic t-15t0 (r(117)=0.24, p=0.009), ∆Diastolic t-15t0 (r(117)=0.31, p=0.001), and ∆HR t-15 t 0 (r(112)=0.22, p=0.020). Although there was no control condition for the trauma-film, these analyses were generally consistent with a time-limited stress response (increase in physiological and subjective arousal/negative affect) in response to the film, followed by recovery within an hour.

Most items on the Bodily Symptoms Scale (BSS, [16]) showed either a V-shaped time (t-15, t0, t+60) effect (but no drug effects), with lowest scores post-film on positively valenced or low arousal states/attributes (i.e. at t0; euphoria, drowsiness, concentration, main effects of time: ps≤0.003) or an inverted V-shaped effect of time (but no drug effects), i.e. peaking at t0 for negatively valenced or high arousal states (anxiety, depression, palpitations, nausea, emotional numbness, muscle tension, tremor; main effects of time: ps<0.001). The 'memory impairment' item of the BSS showed a Time x Drug interaction. However, this reflecting a non-significant (post hoc p=0.102) 2-point larger increase (on a 0-100 scale) between t-15 and t0 in the placebo versus hydrocortisone group. Confusion, headache and vertigo showed no time or drug effects (p values of main effects and interactions ≥0.150). 

Salivary hormones: cortisol and cortisone
Drug and Time effects on cortisol and cortisone levels are shown in Table S1 

Table S1. Cortisol and cortisone levels across four timepoints of the lab session (c.f. three timepoints above; see also Figure 1 and main body of text for explanation). Values are means + SDs.

Cortisol (nmol/L)	t-15	t0	t+15	t+60	
Placebo (n=50)	2.99 (2.94)	10.99 (28.20)	4.41 (5.15)	2.97 (3.59)	
Hydrocortisone (n=47)	3.59 (2.27)	22.31 (19.88)	5.95 (4.19)	86.46 (73.56)	
					
Cortisone (ng/ml)					
Placebo (n=50)	4.69 (2.33)	3.52 (1.18)	4.66 (2.66)	4.36 (2.73)	
Hydrocortisone (n=47)	5.43 (2.08)	3.85 (1.62)	4.70 (2.08)	28.35 (16.23)	
					

Salivary hormones: estradiol and progesterone levels in men and women

Baseline estradiol levels were 3.53 ± 1.47 (range: 0.59-6.70) pg/ml in men and 3.32 ± 1.83 (range: 0.11-8.26) pg/ml in women (t(113)=0.663, p=0.509). Baseline progesterone levels were 8.38 ± 11.13 (range: 0.00-64.90) pg/ml in men and 12.99 ± 17.26 (range: 0.00-72.43) pg/ml in women (t(110)=1.68, p=0.095).  Among women, n=17 used a progesterone-type, and n=42, a combination-type (estrogen + progesterone) contraceptive. Contraceptive type did not affect baseline estradiol or progesterone levels (p values >0.7) or cortisol or cortisone levels (p values >0.4). Other baseline variables summarised in Table 1 also did not differ between women using progesterone-only or combination estrogen-progesterone contraceptive using women (p values ≥ 0.72). 

Intrusive memories across days
Intrusion counts
The regression model used for intrusion counts was:
Intrusions ~ Drug + Day + Drug x Day + (1 | fID)

Summary regression effects from this ZIP GLMM are outlined in Table S2 and estimated marginal means for placebo and hydrocortisone are displayed in Table S3. 


Table S2: Regression coefficient estimates from the ZIP GLMM obtained using the R package, glmmTBM. 
	Estimate	SE	Z value	P(>|z|)	
(Intercept)	1.039243	0.180256	5.765	8.15 ▪ e-09	
Placebo	0.003923	0.231487	0.017	0.98648	
Day 2	h-0.806622	0.129493	-6.229	4.69 ▪ e-10	
Day 3	-1.5388	0.195329	-7.878	3.33 ▪ e-15	
Day 4	-1.95509	0.216973	-9.011	<2.00 ▪ e-16	
Day 5	-2.76308	0.322534	-8.567	<2.00 ▪ e-16	
Day 6	-3.20159	0.379329	-8.44	<2.00 ▪ e-16	
Day 7	-3.31531	0.404417	-8.198	2.45 ▪ e-16	
Placebo: Day2	0.426168	0.180698	2.358	0.01835	
Placebo: Day3	0.585453	0.248312	2.358	0.01839	
Placebo: Day4	0.754535	0.294237	2.564	0.01034	
Placebo: Day5	0.904374	0.400667	2.257	0.024	
Placebo: Day6	1.484992	0.442473	3.356	0.00079	
Placebo: Day7	1.252689	0.485635	2.579	0.00989	


Table S3: EMMs for placebo and hydrocortisone Note the EMMs are in log rather than response units.

Placebo					
Day	Est marginal mean	SE	95% CI (lower)	95% CI (upper)	
1	1.0432	0.165	0.72	1.3665	
2	0.6627	0.176	0.317	1.0083	
3	0.0898	0.2	-0.302	0.4816	
4	-0.1574	0.238	-0.624	0.3089	
5	-0.8155	0.274	-1.354	-0.2768	
6	-0.6734	0.265	-1.193	-0.1537	
7	-1.0195	0.303	-1.615	-0.4242	

Hydrocortisone					
Day	Est marginal mean	SE	95% CI (lower)	95% CI (upper)	
1	1.0392	0.18	0.685	1.3931	
2	0.2326	0.194	-0.148	0.6133	
3	-0.4996	0.241	-0.972	-0.0267	
4	-0.9159	0.259	-1.424	-0.4075	
5	-1.7238	0.353	-2.418	-1.0301	
6	-2.1623	0.405	-2.958	-1.3665	
7	-2.2761	0.43	-3.119	-1.4327	


Response units can be recovered from the logarithmic values using the equation:

Where EMM = estimated marginal means from table S3 and ð = constant representing the probability of a false zero (here ð =0.2845).
As such (for example):
Day 1 count values in the placebo and hydrocortisone groups were:
Intrusions count Day1,Placebo = 
Intrusions count Day1,Hydrocortisone = 
Day 2 counts were
Intrusions count Day2, Placebo =
Intrusions count Day1, Hydrocortisone = 
Etc. for subsequent days.
These values correspond to the predicted intrusions on days 1 and 2 in Figure 2 in the main paper. 

END OF SUPPLEMENTARY METHODS & RESULTS


Supplemental references
1.	Armbruster DC, Kirschbaum C, Strobel A. The not-so-bitter pill: effects of combined oral contraceptives on peripheral physiological indicators of emotional reactivity. Hormones Behav. 2017; 94: 97-105.

2.	Soni M, Curran HV, Kamboj SK. Identification of a narrow post-ovulatory window of vulnerability to distressing involuntary memories in healthy women. Neurobiol Learn Mem. 2013; 104: 32-8.

3.	Merz CJ, Wolf OT, Sex differences in stress effects on emotional learning. J Neurosci Res. 2017; 95(1-2): 93-105.

4.	Holmes,EA, et al. Can playing the computer game “Tetris” reduce the build-up of flashbacks for trauma? A proposal from cognitive science. PloS One, 2009. 4(1): e4153.

5.	Kamboj SK, et al., Reduction in the occurrence of distressing involuntary memories following propranolol or hydrocortisone in healthy women. Psychol Med, 2020. 50(7): 1148-55.

6.	Joëls M, Functional actions of corticosteroids in the hippocampus. Eur J Pharmacol. 2008; 583(2-3): 312-21.

7.	Das R, et al., Nitrous oxide speeds the reduction of distressing intrusive memories in an experimental model of psychological trauma. Psychol Med. 2016. 46(8): 1749-1759.

8.	Watson D,  Clark LA, Tellegen A, Development and validation of brief measures of positive and negative affect: the PANAS scales. J Pers Soc Psychol. 1988; 54(6):1063.

9.	Debono M, et al. Salivary cortisone reflects cortisol exposure under physiological conditions and after hydrocortisone. J Clin Endocrinol Metabol, 2016. 101(4): 1469-77.

10.	Gao WT, Stalder T, Kirschbaum C, Quantitative analysis of estradiol and six other steroid hormones in human saliva using a high throughput liquid chromatography–tandem mass spectrometry assay. Talanta. 2015; 143: 353-8.

11.	Walker JJ, Terry JR, Lightman SL. Origin of ultradian pulsatility in the hypothalamic–pituitary–adrenal axis. Proc Royal Soc B: Biol Sci. 2010; 277(1688): 1627-33.

12.	Lass-Hennemann J, Michael T. Endogenous cortisol levels influence exposure therapy in spider phobia. Behav Res Ther. 2014; 60: 39-45.

13.	Kamboj SK, et al. Ultra-brief mindfulness training reduces alcohol consumption in at-risk drinkers: A randomized double-blind active-controlled experiment. Int J Neuropsychopharmacol. 2017; 20(11): 936-47.

14.	Hartig F. DHARMa: Residual Diagnostics for Hierarchical (Multi-Level/Mixed Regression Models). R package version 0.4.3. 2021.

15.	Brooks ME, et al. glmmTMB balances speed and flexibility among packages for zero-inflated generalized linear mixed modeling. R Journal. 2017. 9(2): 378-400.

16.	Bond A, Lader M. The use of analogue scales in rating subjective feelings. Brit J Med Psychol. 1974. 47(3):  211-18.
17. 	Antony JW, Ferreira CS, Norman KA, Wimber M. Retrieval as a fast route to memory consolidation. Trends Cogn Sci. 2017; 21(8): 573-6.
